# *K*-mer Content, Correlation, and Position Analysis of Genome DNA Sequences for the Identification of Function and Evolutionary Features

**DOI:** 10.3390/genes8040122

**Published:** 2017-04-19

**Authors:** Aaron Sievers, Katharina Bosiek, Marc Bisch, Chris Dreessen, Jascha Riedel, Patrick Froß, Michael Hausmann, Georg Hildenbrand

**Affiliations:** 1 Kirchhoff-Institute for Physics, Heidelberg University, INF 227, 69117 Heidelberg, Germany; Sievers_Aaron@web.de (A.S.); KatharinaBosiek@gmx.de (K.B.); MarcBisch@gmx.de (M.B.); chrisdreessen@yahoo.de (C.D.); jaschelite@googlemail.com (J.R.); Fross@stud.uni-heidelberg.de (P.F.); hausmann@kip.uni-heidelberg.de (M.H.); 2 Department of Radiation Oncology, Universitätsmedizin Mannheim, Medical Faculty Mannheim, Heidelberg University, Theodor-Kutzer-Ufer 1-3, 68167 Mannheim, Germany

**Keywords:** *k*-mer, *k*-mer analysis, sequence analysis, alignment-free, positional features

## Abstract

In genome analysis, *k-mer*-based comparison methods have become standard tools. However, even though they are able to deliver reliable results, other algorithms seem to work better in some cases. To improve *k*-mer-based DNA sequence analysis and comparison, we successfully checked whether adding positional resolution is beneficial for finding and/or comparing interesting organizational structures. A simple but efficient algorithm for extracting and saving local *k*-mer spectra (frequency distribution of *k*-mers) was developed and used. The results were analyzed by including positional information based on visualizations as genomic maps and by applying basic vector correlation methods. This analysis was concentrated on small word lengths (1 ≤ *k* ≤ 4) on relatively small viral genomes of *Papillomaviridae* and *Herpesviridae*, while also checking its usability for larger sequences, namely human chromosome 2 and the homologous chromosomes (2A, 2B) of a chimpanzee. Using this alignment-free analysis, several regions with specific characteristics in *Papillomaviridae* and *Herpesviridae* formerly identified by independent, mostly alignment-based methods, were confirmed. Correlations between the *k*-mer content and several genes in these genomes have been found, showing similarities between classified and unclassified viruses, which may be potentially useful for further taxonomic research. Furthermore, unknown *k*-mer correlations in the genomes of Human Herpesviruses (HHVs), which are probably of major biological function, are found and described. Using the chromosomes of a chimpanzee and human that are currently known, identities between the species on every analyzed chromosome were reproduced. This demonstrates the feasibility of our approach for large data sets of complex genomes. Based on these results, we suggest *k*-mer analysis with positional resolution as a method for closing a gap between the effectiveness of alignment-based methods (like NCBI BLAST) and the high pace of standard *k*-mer analysis.

## 1. Introduction

In recent years, *k*-mer-based analysis and comparison methods have become standard tools for the analysis of large DNA sequences such as chromosomes, whole genomes, or even metagenomes. The big advantage of *k*-mer-based methods compared to alignment-based methods such as the well-established National Center for Biotechnology Information (NCBI) Basic Local Alignment Search Tool (BLAST) software family [1], is the shorter computation times or more precisely, the better scaling of computation times with sequence length [2].

For many purposes, standard *k*-mer methods deliver reliable results. However, there are also cases where they are still very unsatisfying when compared with those of other methods, for example, during the determination of the phylogenetic distance between two genomes, where the alignment of short DNA motifs such as highly conserved ribosomal RNA genes delivers very reliable results, while the results of *k*-mer methods are often uncertain [3]. Perhaps the main difference between the results of an alignment-based and *k*-mer-based method when used on the same data set (e.g., comparison of two genome sequences), is that the results of the alignment method can include the exact position (in bp) and quality of similarity of every part of the sequence within the data set. In contrast, the standard *k*-mer-method only interprets the sequences as “bags of words”, therefore neglecting any positional information [4]. The result of a standard *k*-mer analysis is mostly only comprised of a number representing the similarity between each pair of sequences.

To improve the results of *k*-mer methods, it seems reasonable to add positional resolution to the analysis, while maintaining the advantage of a faster computation time. Algorithms performing such a local *k*-mer analysis have been developed and published [5,6]. A mapping of local *k*-mer spectra on chromosomes (called “genomic barcode”) was used to successfully correlate uncommon (with respect to the rest of the sequence) local *k*-mer structures with regions underlying horizontal gene transfer [5]. In another study, the coding and non-coding parts of the genome were compared between and inside of different organisms [6]. A correlation of local sequence features and *k*-mer-related data (namely local A/T ratio) was accomplished for parts of smaller eukaryote genomes [7]. Beside these positive prior results, it is a long-known fact that certain DNA motifs of major scientific interest, like pathogenicity islands [8], target regions for gene silencing [9], low complexity regions [7], non-globular domains [10], transposons, or simply genes in general [11], are associated with peculiar local G+C content or monomer contents, respectively. Knowing this, we ask two questions in this paper. First: Is it possible to use local *k*-mer analysis results to effectively detect local DNA features formerly only visible through synteny- or alignment-based methods, or even those which are completely unknown? Second, since global *k*-mer features are known to be evolutionary conserved [12]: Is it possible to correlate the presence, position, or characteristics of such features with evolutionary constraints, in order to show whether they are evolutionary conserved?

## 2. Materials and Methods

For our analysis, we used publicly available unmasked nucleotide sequences from the NCBI website in FASTA format (Table 1) [13]. In some cases, data in the GenBank format were also used, if a comparison with gene positions was required. Unmasked versions were used because one of the main goals was to limit prior computation and the use of prior knowledge. This allows also the identification of DNA features like low complexity regions, which are known to show peculiar *k*-mer patterns in some organisms [7] and which would most likely be affected by the masking of, e.g., repetitions.

### 2.1. K-*mer* Analysis

In this article, a *k*-mer analysis of a DNA sequence is considered as the extraction and counting of every DNA word with length *k* (*k* bases along one strand), using a “sliding window” approach [4] to eliminate the influence of an arbitrary chosen starting point. Therefore, we extract one word for every position within the analyzed sequence. We have chosen word lengths of 1 ≤ *k* ≤ 4 due to the fact that many monomer (*k* = 1) features were well described, and because *k* = 4 seems to be a reasonable limit to produce low computation times while preventing a bias resulting from the influence of a potentially underlying amino acid code (for *k* = 3). We generated results for a range of *k* (1 ≤ *k* ≤ 4), instead of just using, e.g., *k* = 4, because we wanted to analyze whether a detected feature is only present for a certain value of k or for a range of different values of k. This could provide insights into the word lengths responsible for generating these features, which could lead to different interpretations.

The result of such a *k*-mer analysis of a single sequence, meaning the frequencies/contents of each DNA word of length k, is called the associated “*k*-mer spectrum”. Such a *k*-mer spectrum can also be interpreted as a 4 *k* dimensional vector, and as such, vector distances can be applied to compare two or more *k*-mer spectra, in order to calculate a value which can be interpreted as a measurement of similarity between associated DNA sequences. For a better normalization, the Pearson correlation function [14] was used over the Euclidean distance. Using the Spearman correlation function to prevent issues due to extreme values/contents did not result in any significant advantages. The Pearson correlation function is a vector difference mapped to values between −1 and +1, where +1 stands for a perfect correlation and −1 is a perfect anticorrelation. Accordingly, values near one were considered as a “good/high” correlation and values near zero as a “bad/low” correlation.

For the allocation of the individual *k*-mers, the bp position numbers (of the first base of each word) in the respective genomes were used. To prevent confusion when DNA words of different lengths are discussed, we will always use a slash if two (or more) separated sequences are meant. For example, “A/C” means the two different lengths of the *k* = 1 words with one “A“ and “C”. “AC” means the single *k* = 2 word, consisting of one “A”, followed by one “C”.

### 2.2. Local k-*mer* Analysis

To obtain a local *k*-mer spectrum from a large DNA sequence, a two-step approach was applied. First, we wrote an efficient program in C/C++ to save the positions of every single *k*-mer of a given length *k* using a hash map (using the DNA words as keys for the mapping). Our program saves the positions in a binary file and simultaneously creates an index structure for fast access at a later point in time. In a second step, a simple binning algorithm discretized our data by position. In the end, the results are equivalent to cutting the whole sequence into segments of equal length (corresponding to the bin width) and performing a *k*-mer analysis as described above for each of the resulting segments/cuts. We use this two-step approach instead of a more direct method because it allows us to generate results for different segment sizes (respectively bin widths) more effectively, by saving intermediate results to a hard disc.

The computation times of other *k*-mer tools, such as JellyFish [15], are not comparable to our tool, because we need to use a more complex algorithm to perform a local *k*-mer analysis (especially concerning writing operations to store the position information). However, the analysis of large chromosomes (e.g., *Homo Sapiens* c2) is possible in less than 20 min on ordinary desktop machines.

### 2.3. Relative Spectra

We created artificial spectra with a DNA word length of *k* + 1, based on the extracted spectra with word length *k* using Zero-Order Markov models for *k* = 1 and higher order Markov chain models for *k* > 1.

By dividing the frequencies of the extracted spectra by the corresponding values of the artificially created spectra with the same word length *k*, one obtains relative spectra where the influence of the *k* = 1 frequencies, e.g., the influence of the G/C-content for *k* = 2, is removed. A similar method was developed by [16]. In the following, such spectra will be called “relative spectra” and their DNA word contents will be known as “relative contents”. If the spectra or DNA words without the prefix “relative” are mentioned, we always refer to the directly extracted spectra/words. A relative analysis separates the visible features obtained by a word length *k* from features originating from a word length *k* = 1. For example, the relative *k* = 3 spectrum shows additional information to the *k* = 2 spectrum, because it was corrected for any trivial correlations.

### 2.4. Mapping

The results of a local *k*-mer analysis were visualized by mapping comparable to the method of [5]. A linear representation of the sequence from 5′ at the top, to 3′ at the bottom, was set up (even if some analyzed genome sequences are circular molecules in reality), with a column for each DNA word (sorted alphabetically). A linear mapping correlating color and *k*-mer frequency were used. White corresponds to the minimum value and a clear/pure color to the maximum value found within the bins of the local *k*-mer analysis (see Section 2.2). Using this approach, we ensure to always exploit the maximum color range, and thus, the maximum color resolution available. To produce quantitative results concerning such a feature, we used the visualization to identify and localize particular features. This means that we always extracted and analyzed the exact local *k*-mer spectrum of a specified region, even if we do not always explicitly show the associated data.

### 2.5. Correlation Heatmaps and Mean Correlations

The results of a local *k*-mer analysis of two or more sequences (e.g., genomes or parts of genomes) can be seen as a list of *k*-mer spectra, ordered by their position inside of the sequence. The Pearson correlation function, as mentioned above, was used to calculate a correlation value between each single (local) *k*-mer spectrum of one set with each *k*-mer spectrum of the other set (or number of sets). The result is a two-dimensional matrix-like structure, where each row is associated with a region in one sequence (more precisely, with a bin associated with a certain region) and each column is associated with a position in the other sequence (or sequences). Similar structures were analyzed by [6]. Such a matrix can be displayed as a heatmap, by mapping the correlation value to a color scale. An area inside of such a heatmap is then equivalent to the correlation of local *k*-mer structures.

The mean values of such areas or complete heatmaps were taken to quantify the level of correlation between specific regions, while the standard deviation was used for an error estimation. Two mean values with overlapping error ranges, however, were not assumed to be significantly different in a strict statistical sense. For example, comparing a mean value of 0.75 ± 0.5 with a mean value of 0.99 ± 0.01 would not be significantly different in the given error ranges. However, referring to the relative accuracy of the given data indicates a huge difference in the spread of the analyzed data sets.

For creating the image files (not only the maps and heatmaps), we used simple python scripts written by the authors, using the well-known matplotlib library for Python 2.7 [17].

### 2.6. Heatmap Summary Images

Since showing many such heatmaps is not suitable, and since heatmaps are often hard to interpret and compare, we use a visualization method producing “heatmap summary images”. To generate these images, we took the mean values and standard deviations of the correlation values in the heatmaps (see Section 2.5), or of interesting areas within the heatmaps. We generated an image with these mean values as data points and the standard error of this mean value (derived from the standard deviation) as error bars. Therefore, we created an easy to compare, two-variable summary for many heatmaps within one image.

We should mention that the correlation values do not necessarily follow a normal distribution, and therefore, such a representation is not guaranteed to be a good summary of heatmap data, but since it objectively reproduces our observations made within the heatmaps, it seems to be a reliable and objective method.

### 2.7. Software

All of the codes and scrips (including visualization) used for this article, as well as an English manual, are freely available online at http://www.kip.uni-heidelberg.de/biophysik/software.

## 3. Results

### 3.1. Viral Genomes

As a proof of principle to our approach, several viral genomes were chosen. Viral genomes are generally very short and therefore very fast to analyze using our *k*-mer algorithms. They contain only a small absolute number of genes and other DNA motifs, while nearly their complete genomic sequences hold known and well-understood biological functions. This allows us to verify the results of a local *k*-mer analysis, as well as to gain new insights into the currently unknown functional correlations of characteristics of *k*-mer spectra.

#### 3.1.1. *Papillomaviridae*

Human Papillomavirus (HPV) is a double stranded nonenveloped circular DNA virus. We analyzed and compared genomic sequences of 11 different HPV types covering a wide phylogenetic range [18], also including some very close relatives.

**Figure 1:**
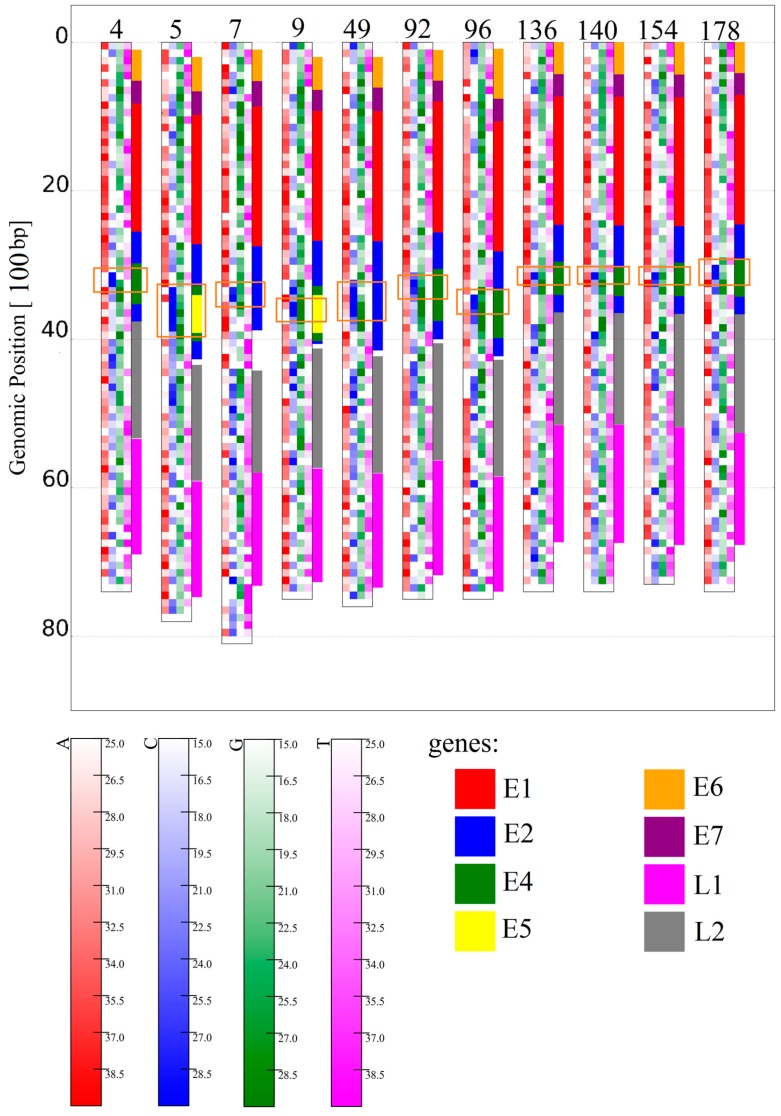
Map of Human Papillomaviruses (HPVs) for *k* = 1. From left to right: HPV4, 5, 7, 9, 49, 92, 96, 136, 140, 154, 178. A bin width of 100 bp was used. Genes were represented by colored bars at the right side of the linear representation of the circular HPV genomes (E1 red, E2 blue, E4 green, E5 yellow, E6 orange, E7 purple, L1 magenta, L2 grey). The orange boxes indicate the boundaries of the three regions with different *k*-mer structures (the regions above and below the middle region in the box do not have their own boxes for easier readability).

**Figure 2:**
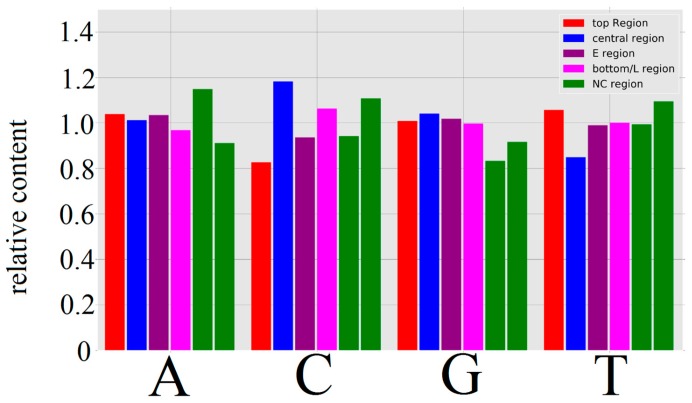
Histogram of monomer contents of different regions relative to the monomer content of the whole genome of HPV4. The top region (**red**) is associated to the genes E1, E6, and E7. The central region (**blue**) is associated with E2, E4, and E5. The E region (**purple**) is the region covered by all early genes. The bottom/L region (**magenta**) is associated with the late genes (L1, L2). The two NC regions (**green**) on the left and right are the non-coding region at the top and bottom of the linear representation used in Figure 1, respectively.

All HPV genomes analyzed show at least three regions with distinguishable *k*-mer structures (Figure 1). For an analysis of these regions, the content of C and T were chosen as criteria. The first region is then located at the top ~40% of each genome showing a relatively low C content (~20% lower than HPV average), associated with the genes labeled E1, E6, and E7 for each HPV genome shown (Figure 2). This is followed by a relatively small region (~10%–20% in size) at the center of each genome showing a very high content of C (~20% over HPV average) and low content of T (~18% lower than HPV average) associated with the genes E2, E4, and E5 for every HPV type shown. Lastly, a large region (~40% in size) with a slight increase in the C content, but otherwise without clear monomer preferences located at the bottom, is associated with the late genes (L1, L2). For higher word lengths (1 ≤ *k* ≤ 4), the stated regions are also visible and show similar word contents for all HPV types (Figures S1 and S2).

**Figure 3:**
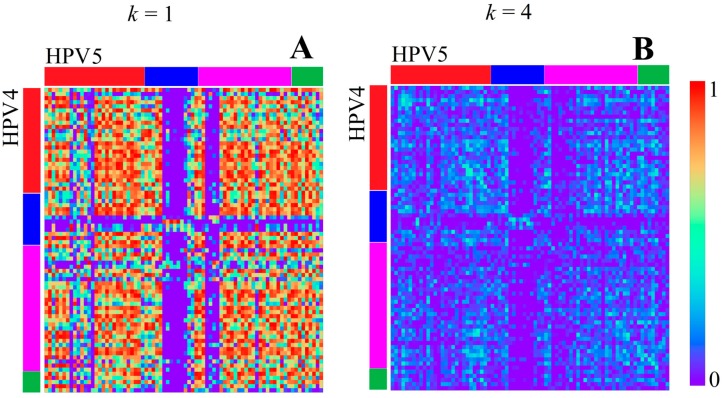
Correlation heatmaps between HPV4 and 5 for *k* = 1 (**A**) and *k* = 4 (**B**) (bin width of 100 bp). The colored bars at the edges indicate the locations of the top region (**red**), central region (**blue**), bottom region (**magenta**), and NC region (**green**) according to gene annotation borders (not by *k*-mer content).

**Figure 4:**
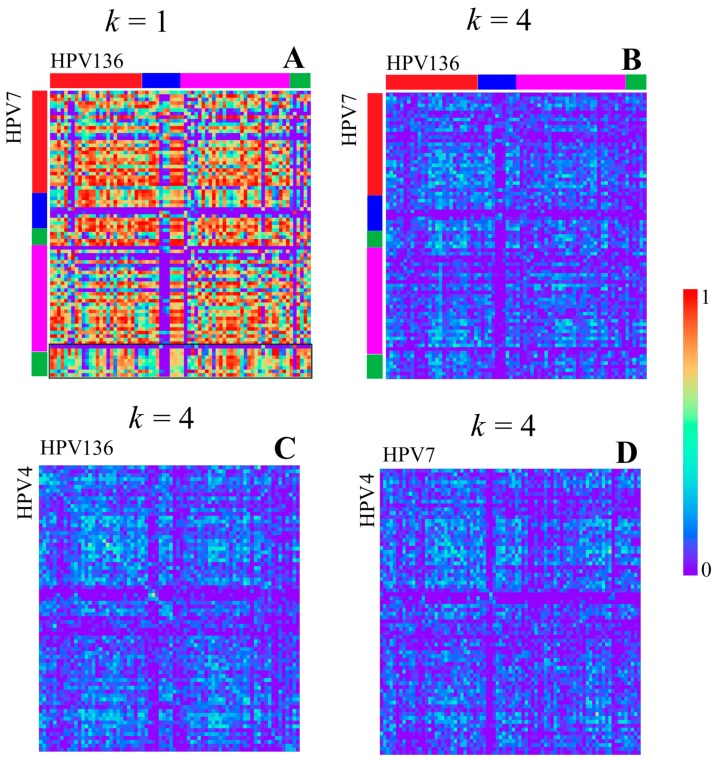
Correlation heatmaps of different HPV types for different *k* values (*k* = 1 in **A**+**C**, *k* = 4 in **B**+**D**), with a bin width of 100 bp. The colored bars at the edges indicate the positions of the top region (red), central region (blue), bottom region (magenta), and NC region (green) according to the borders of associated genes (not by *k*-mer content). The peculiar structure of the NC region of HPV 7 is highlighted with a green border in (**A**, *k* = 1). The linear structures between HPV4 and HPV136 are considered as “strong” between HPV7 and HPV4, and are “weak” between HPV7 and HPV136.

**Figure 5:**
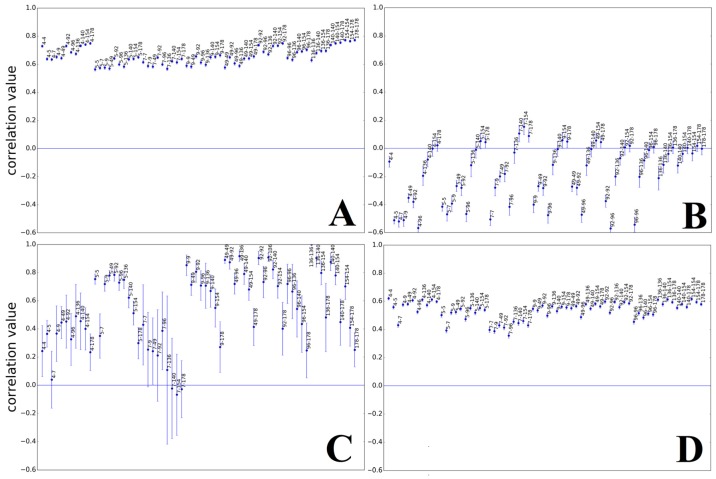
Heatmap Summary Images of HPV Regions. Shown are the summaries of some heatmaps (*k* = 1) of the three regions defined by different *k*-mer contents for all of the HPV types analyzed (positions of regions can be found in Table S1). (**A**) Good correlation within the first region between all HPV types; (**B**) Bad correlation (values around zero or lower) between the first and second region; (**C**) Relatively good correlation in the second region for most values; (**D**) Good correlation amongst all of the third regions of HPV. The data points are equally spaced and sorted numerically, therefore no additional information is provided by their horizontal alignment. The labels next to the data points indicate the corresponding HPV types whose regions were correlated.

The local mean correlation values between HPV types support the impression of distinctive *k*-mer structures inside each of the described regions (e.g., Figure 3 shows a correlation heatmap between HPV 4 and HPV 5). According to the *k*-mer content in the map of Figure 1 and heatmap values of Figure 3 and Figure 4, the relation between the top (E1, E6, E7) and bottom region (L1, L2) is dominated by good correlation values, while the central region (E2, E4, E5) is well recognizable by a bigger region of low correlation with any of the other two. These observations are supported and displayed for all of the HPV types in Figure 5. The high correlation values (0.6–0.9) in Figure 5A,D confirm that the high similarity within the first and third identified region is present for, and also between, all HPV types analyzed, respectively. The presence of the low correlation between the first and the second region is confirmed for and between all HPV types by the relatively low values (−0.5–0.1) displayed in Figure 5B. The correlation within the second region (see Figure 5C) is not as high as within the first or third region, but is still significantly higher than between the first and second region for most HPV types.

This means that the gene-related regions described in [19] are visible through *k*-mer features. The relations between their *k*-mer structures differ from the relations between the genes when seen under the aspect of early and late genes. It is also remarkable that the central C rich region is the only region containing overlaps of a significant size between genes (in a range from 28% up to 100% for E2, E4, and E5, whereas all other genes are below 10%, and most of them are below a 5% overlap). This could mean that the identified *k*-mer features are not directly associated with the time of gene activity, but with the gene density or gene overlap.

The longer we set the word length *k*, the more extreme the correlation values become. This means, for example, for *k* = 4, only very similar regions show a good correlation, while the mean correlation value becomes almost zero (visible in Figure 3 and Figure 4). Therefore, we found a feature for *k* = 4 that was not visible at a smaller *k* value, namely a slightly better correlation of a linearly distributed number of bins, e.g., visible between HPV 4 and HPV 5 as diagonal elements in Figure 3B. This linear structure is equivalent to the fact that the linear positional distribution pattern of *k*-mers is conserved between two sequences, and is therefore related to similar sequences of DNA words and probably related to a good alignment result. Such linear structures are existent in any HPV genome analyzed. It is remarkable that the linear structure is the weakest for any correlation of the genomes with HPV7, which is the only analyzed virus classified as *Alpha Papillomaviridae*. This may represent the evolutionary distance to all other types, classified as *Beta* and *Gamma Papillomaviridae*. All of the *Beta Papillomaviridae* (HPV 5, HPV 9, HPV 49, HPV 92, and HPV 96) show a strong correlation among themselves and a similar but slightly weaker correlation with HPV 4, the only *Gamma Papillomaviridae*. HPV 4 itself shows a very strong linear structure when correlated with any of the unclassified species HPV 136, HPV 140, HPV 154, and HPV 178 (examples given in Figure 4). All unclassified species show strong linear structures when compared to each other or HPV 4. Slight correlations might be found if HPV 136 and HPV 140 are seen in comparison with HPV 92. This could mean that the unclassified (with respect to HPV subphyla) HPV types are not related to HPV 7 and are presumably more linked in the subphylum of *Gamma Papillomaviridae*, namely HPV 4, than in the *Beta Papillomaviridae*.

#### 3.1.2. *Herpesviridae*

As a second group of representatives, we chose a wide phylogenetic range of the Human Herpesvirus (HHV) types [20]. HHV contains an enveloped linear genome and is well known to have one of the largest and most complex genomes of all viruses.

**Figure 6:**
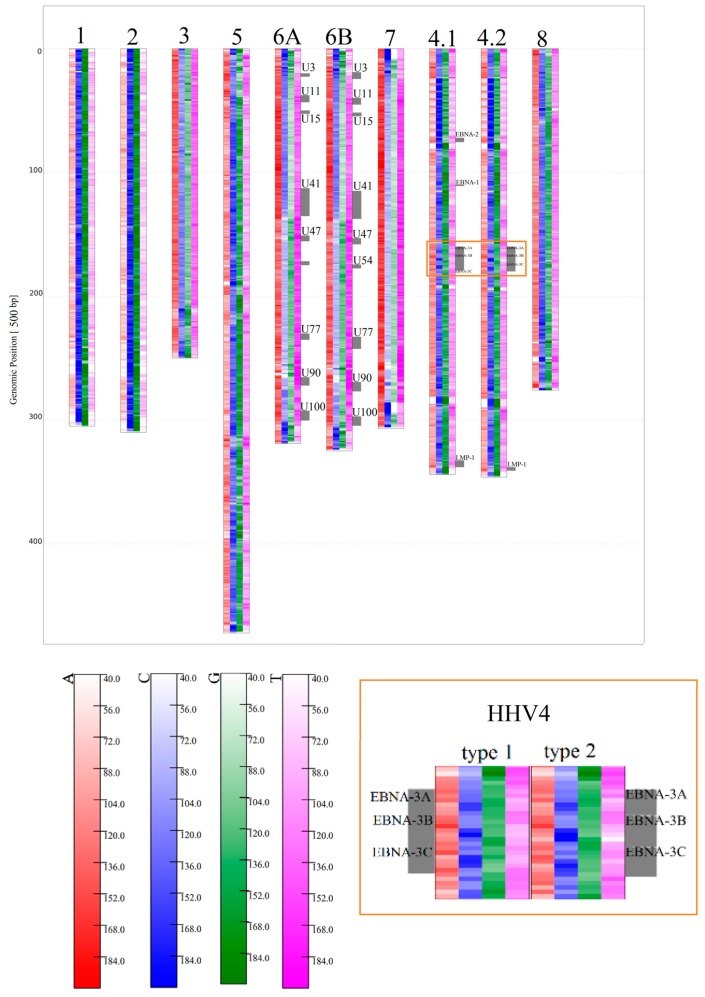
Map of Human Herpesvirus (HHV) genomes for *k* = 1, with a bin width of 500 bp. From left to right: HHV1, 2, 3, 5, 6A, 6B, 7, 4 type 1, 4 type 2, 8. Genes associated with low conserved regions on HHV6A, 6B, 4 type 1, and 4 type 2 were visualized with gray bars at the side of the genomes. At the right bottom corner, a small region around the EBNA genes for both HHV4 species is shown to illustrate small differences in the local *k*-mer structure.

**Figure 7:**
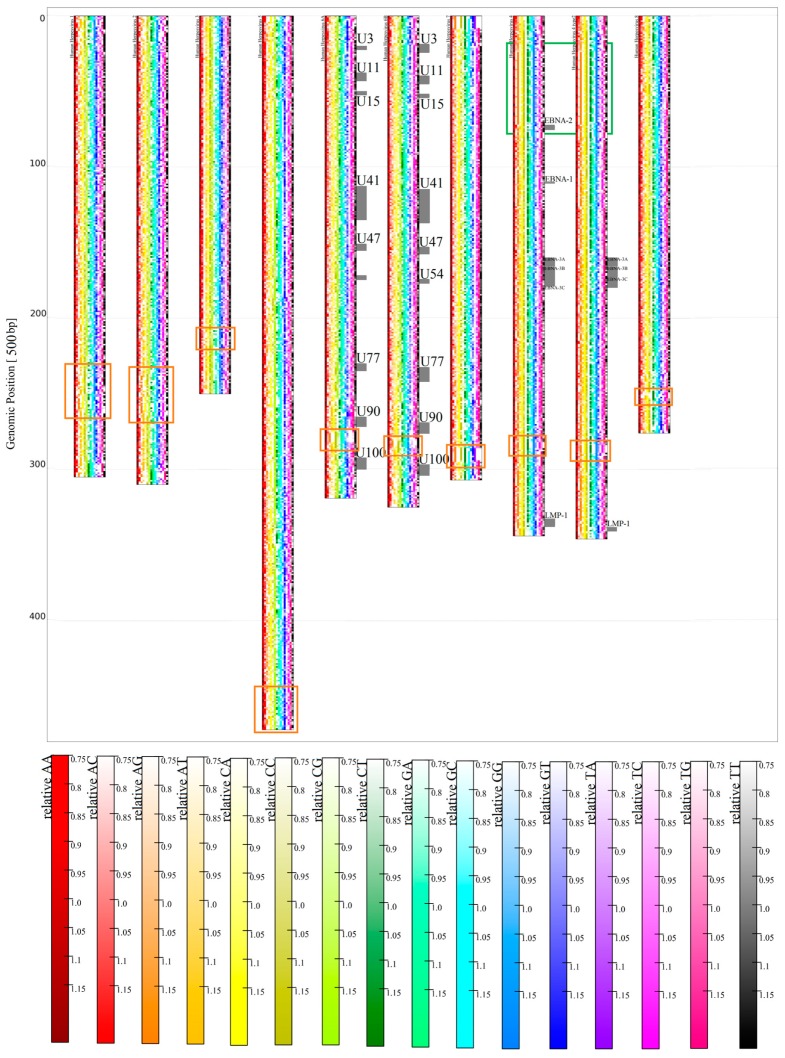
Map of HHV genomes for relative *k* = 2, with a bin width of 500 bp. From left to right: HHV1, 2, 3, 5, 6A, 6B, 7, 4 type 1, 4 type 2, 8. Peculiar patterns associated with high C or C/G content for *k* = 1 are marked orange, and the iterative structure at the top of 6A and 6B is marked green.

The analyzed HHV types cover a relatively wide range of global G/C contents, from G+C > 64% for HHV1 and HHV2 to < 40% for HHV7. All of the HHV genomes analyzed obey Chargaff's second rule [21] on a global scale. There is a clear tendency for higher C/G values at a region near to, but not necessary always directly at, the ends and the beginnings of all HHV genomes. Moreover, every HHV type seems to have a small part, 1500 bp–15,000 bp, with an extremely high C and/or G content (low A/T content respectively) in the 3’ part, accounting for 10%–25% of their genomes (Figure 6). The local contents of G/C and A/T, especially in these regions, often clearly violate Chargaff's second rule, therefore justifying the separated treatment of G and C (A/T respectively) for HHV. These regions are also visible for higher *k* values (Figures S3 and S4) and are the only feature clearly visible on relative *k*-mer maps (Figure 7). In contrast to *k* = 1, the features on the relative maps do not share a very uniform structure considering relative *k*-mer contents, except for very close relatives (HHV6A, HHV6B, and HHV4 type 1 and 2).

**Figure 8:**
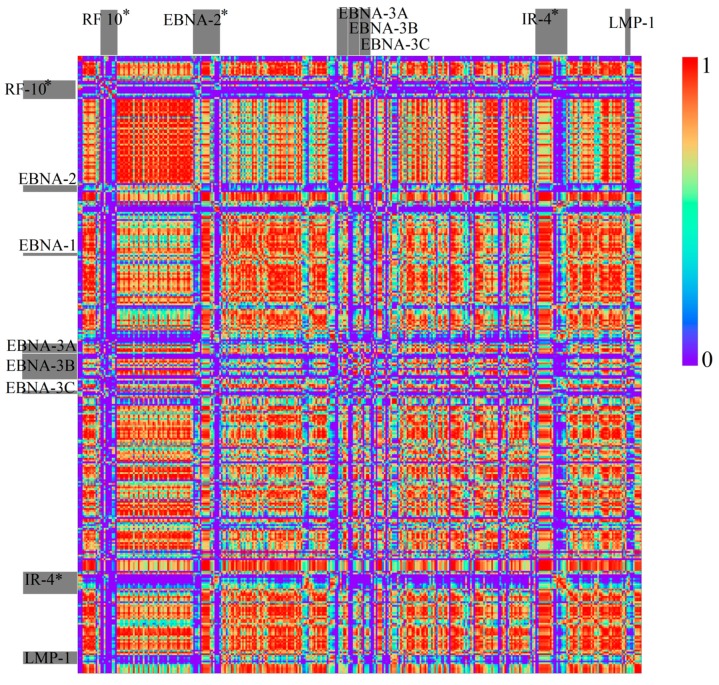
Correlation heatmap between HHV4 type 1 and HHV4 type 2 (*k* = 1 and bin width of 500 bp). The gray bars indicate genes associated with regions of low conservation derived with alignment methods. Genes with an * were not found for the sequences used in our analysis. Therefore, their positions are only approximated by using the data from [23].

**Figure 9:**
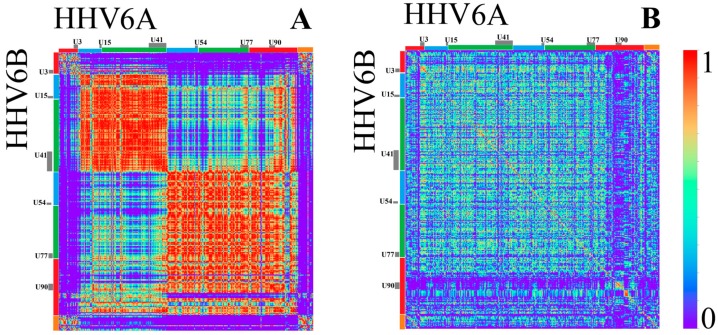
Correlation heatmap between HHV6A and 6B for *k* = 1 (**A**) and relative *k* = 2 (**B**) (bin width 500 bp). Genes are represented by grey bars at the borders. Regions with a low identity score in [22] are marked orange, extremely low values are red, high identity scores are blue, and extremely high values are green.

HHV6A and HHV6B are closely related, but due to differences discovered through alignment methods, are not considered as a single species [22]. Therefore, their genome sequences should not be as similar as the two HHV4 types are. HHV6A and HHV6B show very similar structures in Figure 6 and Figure 7, but are far from identical if compared with the two types of HHV4. Again, this confirms former classifications (see Figure 8 and Figure 11B). A comparison between HHV6A and 6B using an alignment method was made in [22]. They identified regions of extreme low, high, and extremely high conservation, and again associated their location with certain genes (all marked with bars in Figure 9).

When looking at the maps, the *k* = 1 patterns of HHV6A and 6B look quite similar , whereas a correlation visualized in Figure 9 shows that both genome sequences show complex structural relations. At least five different regions with a relative high degree of self-similarity (regions of red in correlation map) become visible for the monomer structures (Figure 9A). The regions at 5′ and 3′ show similarities among themselves, both being present in low conservation regions. The region between U3 and U41 shows a structure clearly distinguishable from the structure between U41 and U90 for HHV6A and 6B. The first region is associated with regions of high identity (blue and green bars) in [22]. The second is also mostly associated with regions of high identify, but also spans over the region of extremely low identity around U90. In the map, this region does not have a very clear representation on a monomer level (Figure 6) and is inhomogenous in its correlation values for *k* = 1. One should also remark that [22] mentioned issues with the alignment in this specific region and therefore changed the parameters of their analysis.

Other than in the monomer structures, relative *k* = 2 structures (Figure 9B) of HHV6A and 6B clearly show differences between low and high conserved regions. The bigger regions, with a relative high self-similarity, seem to fit with regions of a high conservation, to a higher degree. The region around U90 is especially visible a very small area of self-similarity, but does not strictly exhibit a strong relation with any other area of self-similarity. This supports the usefulness of the consideration of relative *k*-mers, while also proving that not all *k*-mer features are simply based on the monomer content (or even C+G content).

**Figure 10:**
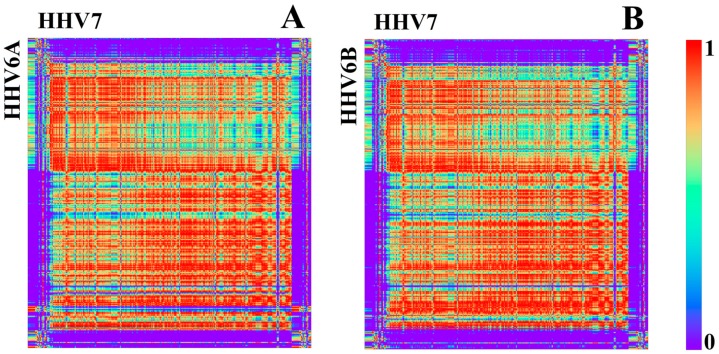
Correlation heatmap between HHV6A with HHV7 (**A**) and HHV6B with HHV7 (**B**) based on *k* = 1 (bin width 500 bp).

**Figure 11:**
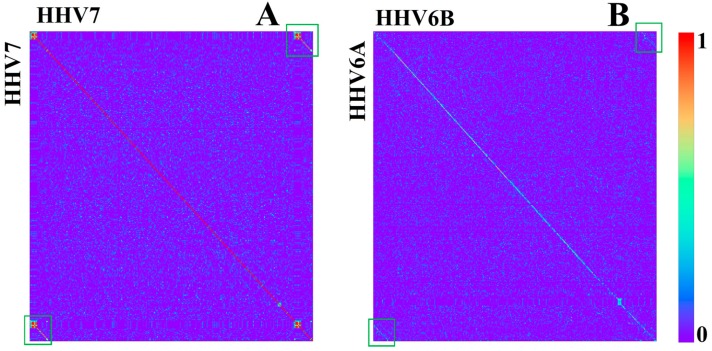
Correlation heatmap between HHV7 with itself (**A**) and HHV6A with HHV6B (**B**) based on relative k = 4 (bin width 500 bp). Beginning and ending regions are highlighted with green boxes.

HHV7 is also a close relative of 6A and 6B [24]. Therefore, it is expected to show similar *k*-mer structures. It was shown in [24] that the differences between 6A, 6B, and 7 are located mainly at the so called “repeat regions” at the beginnings and ends of the genomes. Correlations of *k*-mer structures confirm these results (Figure 10). Besides the fact that the beginnings and ends show low mean conservation values, it is remarkable that their linear relative *k*-mer structure within one genome is strictly conserved when comparing the 3′ region with the 5′ region (Figure 11). This feature is only existent for 6A, 6B, and 7. For 6A and 6B, the structure is also conserved between the two genomes, although this is not true when compared with 7 (Figure 11).

Other than for the analyzed HPV, only a small number of closely related groups of HHV show a conserved overall linear *k*-mer structure (Figure 11). Conserved linear structures were only visible between HHV1 and 2, which have a relation similar to HHV6A/B and 7 [20], between both types of HHV4, and between HHV6A/6B and 7. Most of them are also only clearly visible for *k* = 4. To check if the absence of conserved linear *k*-mer structures depends on the larger bin width, we repeated the analysis with a bin width of 100 bp, without a deviating result.

### 3.2. Homo sapiens

**Figure 12:**
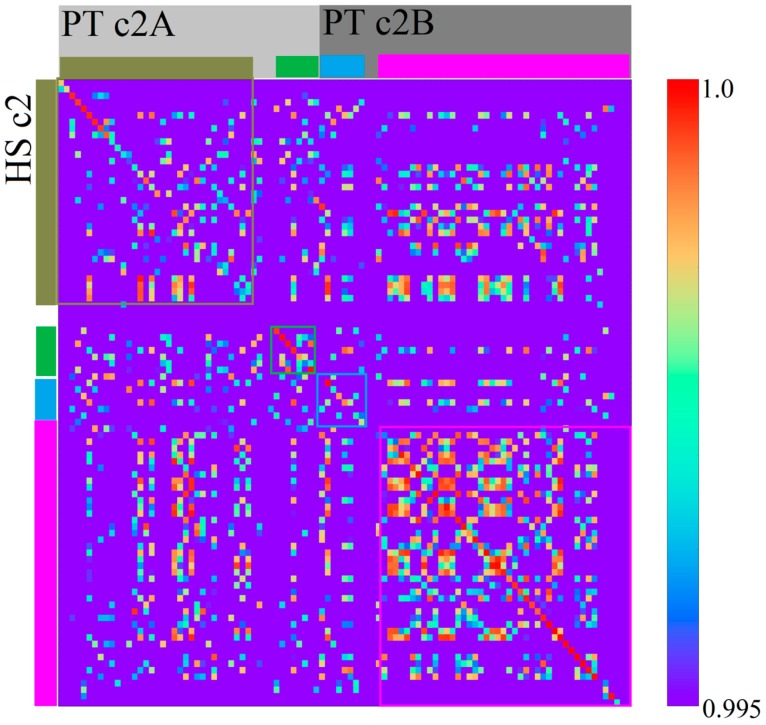
Correlation heatmap between *Homo sapiens* chromosome 2 (HSc2) and *Pan troglodytes* chromosome 2A (PTc2A) and 2B. Based on *k* = 4 (bin width 2.5 Mbp) with a highly increased threshold (see scale on the right). The colored boxes indicate regions of association between the chromosomes.

To check whether our methods are also reliable and efficiently usable on larger scale sequences, we created maps of the relatively large human (*Homo sapiens*) chromosome 2 (HSc2) and chimpanzee (*Pan troglodytes*) chromosomes 2A and 2B (PTc2A, PTc2B respectively). It is a well-known fact that HSc2 is the result of a fusion of chromosomes related to PTc2A and PTc2B of an ancestor of *H. sapiens* [25]. By using our methods on HSc2 and PTc2A/B, a number of linear conserved regions could be identified (Figure 12). We should mention that in comparison to analyzed viral genomes, it was necessary to use a very high threshold for the color scale of the correlation heatmap to recognize linear structures diagonally, even for *k* = 4. This seems mostly based on the fact that we used a relatively large bin width of 2.5 Mbp to create the images. A value of 2.5 Mbp is arguably large enough for a segment to show a specific genomic *k*-mer structure. This effect of a specific genomic *k*-mer structure for segments of sufficient size was formerly described and used in [5]. An association between the human and chimpanzee chromosomal sequences to identify closely related regions was demonstrated in [26]. The regions which exhibited an association between HSc2 and the two PTc2 are visualized in Figure 12 by bars. Boxes based on these bars have been drawn on the correlation heatmap. The linearly conserved regions, identified by our method as diagonals in a correlation heatmap, seem to be comparable to the results of [26]. This illustrates and confirms the functionality of our methods for large sequences.

## 4. Discussion

In this article, two questions were addressed. We showed that local *k*-mer structures correspond to genome feature analysis. We identified several local *k*-mer features representing regions with high or low conservation, as confirmed by independent methods (mostly alignment or gene based). We distinguished the *k*-mer structures of the early and late gene regions for all HPV types and obtained a measure for the quality of conservation between different types of HPV genomes. Expanding our analysis to humans and chimpanzees correctly verified the predicted homologies. The second question focused on whether such features are evolutionary conserved. Regions identified as conserved by other methods were also visible in the *k*-mer-based results and were reasonably associated with phylogenetic distances. Some features (e.g., the linear conservation of the beginning and ending regions in HHV6A/6B and 7), were only visible for close relatives. The conservation of linear structures visible on correlation heatmaps seems to clearly indicate closely related sequence regions. Accordingly, a classification of some unclassified HPV types was feasible. Nevertheless, these results should be verified by other techniques, in order to demonstrate their usefulness for the classification of other genomes without the requirement of prior knowledge (e.g., gene function), which is even more probable for *k*-mer analysis with positional resolution as a whole.

Furthermore, a number of local *k*-mer features were identified without a description or explanation in any publication found, and thus, may not detectable by other methods or only with difficulty. This is the case for the repetitive region at the top of the HPV4 genomes and also for the linearly conserved beginnings and endings of HHV6A/6B and 7. Highly conserved sequences are often an indication of evolutionary pressure and therefore of biological or physical functionality, which leads to the assumption that the identified regions are of high functionality.

The hypothesis that the overall *k*-mer content, especially the content of G/C or local aberrations, from the global *k*-mer structure are involved in, for example, immune evasion, is not new [9,27,28]. Many other interesting genomic features and motifs like codon usage [29], gene length [30], and the distribution and classification of repetitive elements [31], are known to be associated with local G/C content. Higher word lengths k could give different perspectives on certain genomic features or even lead to the detection of currently unknown features. Therefore, *k*-mer analysis with positional resolution might deliver new insights into rules for genome organization, structuring, and evolution.

In summary, *k*-mer analysis with positional resolution, like standard *k*-mer analysis, is very fast, even for sequences far beyond the length scales suitable for alignment methods (e.g., human chromosomes), while generating reliable results even for sequences with a very low level of similarity (where alignment methods often fail). Most of the features detected are not visible by a standard *k*-mer analysis (without positional resolution), since their location and colocalization with other DNA motifs were essential for their detection and identification, and none of them required the detection of secondary biological or other information sources like gene databases. Of course, a local *k*-mer analysis does not outclass every other *k*-mer method and alignment methods like NCBI BLAST remain very powerful tools, but local *k*-mer delivers an interesting additional perspective on sequence data and may close a gap between alignment and alignment-free methods. We believe that further analysis of more DNA sequences and more specific identification and categorization for the cataloging of *k*-mer features might provide new insights into the mysteries of complex genomes.

## Figures and Tables

**Table 1:** Accession numbers of DNA sequences used.

Species	Accession Number
Human Papillomavirus 4 (HPV4)	NC_001457.1
Human Papillomavirus 5 (HPV5)	NC_001531.1
Human Papillomavirus 7 (HPV7)	NC_001595.1
Human Papillomavirus 9 (HPV9)	NC_001596.1
Human Papillomavirus 49 (HPV49)	NC_001591.1
Human Papillomavirus 92 (HPV92)	NC_004500.1
Human Papillomavirus 96 (HPV96)	NC_005134.2
Human Papillomavirus 136 (HPV136)	NC_017994.1
Human Papillomavirus 140 (HPV140)	NC_017996.1
Human Papillomavirus 154 (HPV154)	NC_021483.1
Human Papillomavirus 178 (HPV178)	NC_023891.1
Human Herpesvirus 1 (HHV1)	NC_001806.2
Human Herpesvirus 2 (HHV2)	NC_001798.2
Human Herpesvirus 3 (HHV3)	NC_001348.1
Human Herpesvirus 4 type1 (HHV4 type1)	NC_007605.1
Human Herpesvirus 4 type2 (HHV4 type2)	NC_009334.1
Human Herpesvirus 5 (HHV5)	NC_006273.2
Human Herpesvirus 6A (HHV6A)	NC_001664.2
Human Herpesvirus 6B (HHV6B)	NC_000898.1
Human Herpesvirus 7 (HHV7)	NC_001716.2
Human Herpesvirus 8 (HHV8)	NC_009333.1
*Homo Sapiens* Chromosome 2	NC_000002.12
*Pan Troglodytes* Chromosome 2A	NC_006469.3
*Pan Troglodytes* Chromosome 2B	NC_006470.3

## Supplementary Materials

The following are available online at www.mdpi.com/2073-4425/8/4/122/s1. Table S1: HPV Region Boundaries. Figure S1: Map of HPV viruses for *k* = 2. From left to right: HPV4, 5, 7, 9, 49, 92, 96, 136, 140, 154, 178. A bin width of 100 bp was used. The order of words is alphabetically from left in each genome starting with an AA. Genes were represented by colored bars at the right side of the linear representation of the circular HPV genomes (E1 red, E2 blue, E4 green, E5 yellow, E6 orange, E7 purple, L1 magenta, L2 grey). The box indicates region 2, for borders of all regions see Table S1. Figure S2: Map of HPV viruses for *k* = 3. From left to right: HPV4, 5, 7, 9, 49, 92, 96, 136, 140, 154, 178. A bin width of 100 bp was used. The order of words is alphabetically from left in each genome starting with an AAA. The box indicates region 2, for borders of all regions see Table S1. Figure S3: Map of HHV genomes for *k* = 2, bin width of 500 bp. From left to right: HHV1, 2, 3, 5, 6A, 6B, 7, 4 type 1, 4 type 2, 8. The order of words is alphabetically from left in each genome starting with an AA. Figure S4: Map of HHV genomes for *k* = 3, bin width of 500 bp. From left to right: HHV1, 2, 3, 5, 6A, 6B, 7, 4 type 1, 4 type 2, 8. The order of words is alphabetically from left in each genome starting with an AAA.
