# Supplementary material for: K-mer Content, Correlation, and Position Analysis of Genome DNA Sequences for the Identification of Function and Evolutionary Features
Source: Genes (Basel). 2017 Apr 19;8(4):122. doi: 10.3390/genes8040122 (PMC5406869; doi:10.3390/genes8040122)
Supplement: Supplementary file 1 [file genes-08-00122-s001.zip › Supplemental table.docx]

**Table S1.** HPV Region Boundaries.

| **Species** | **Top Region Boundaries**  **(position in sequence)** | **Central Region Boundaries**  **(position in sequence)** | **Bottom Region Boundaries**  **(position in sequence)** |
| --- | --- | --- | --- |
| Human Papillomavirus 4 (HPV4) | 100 - 2600 | 3100 – 3500 | 3700 – 6900 |
| Human Papillomavirus 5 (HPV5) | 200 – 2800 | 3300 – 3900 | 4300 – 7500 |
| Human Papillomavirus 7 (HPV7) | 100 – 2800 | 3300 – 3500 | 4400 – 7300 |
| Human Papillomavirus 9 (HPV9) | 200 – 2700 | 3500 – 3700 | 4200 – 7300 |
| Human Papillomavirus 49 (HPV49) | 200 – 2700 | 3300 – 3700 | 4200 – 7400 |
| Human Papillomavirus 92 (HPV92) | 100 – 2600 | 3200 – 3400 | 4000 – 7200 |
| Human Papillomavirus 96 (HPV96) | 100 – 2800 | 3400 – 3600 | 4200 – 7400 |
| Human Papillomavirus 136 (HPV136) | 0 - 2500 | 3100 – 3200 | 3600 - 6800 |
| Human Papillomavirus 140 (HPV140) | 0 – 2500 | 3100 – 3300 | 3600 - 6800 |
| Human Papillomavirus 154 (HPV154) | 0 – 2500 | 3100 – 3400 | 3600 - 6800 |
| Human Papillomavirus 178 (HPV178) | 0 - 2500 | 2900 - 3400 | 3600 - 6800 |
