# Supplementary figures and images for: K-mer Content, Correlation, and Position Analysis of Genome DNA Sequences for the Identification of Function and Evolutionary Features
Source: Genes (Basel). 2017 Apr 19;8(4):122. doi: 10.3390/genes8040122 (PMC5406869; doi:10.3390/genes8040122)

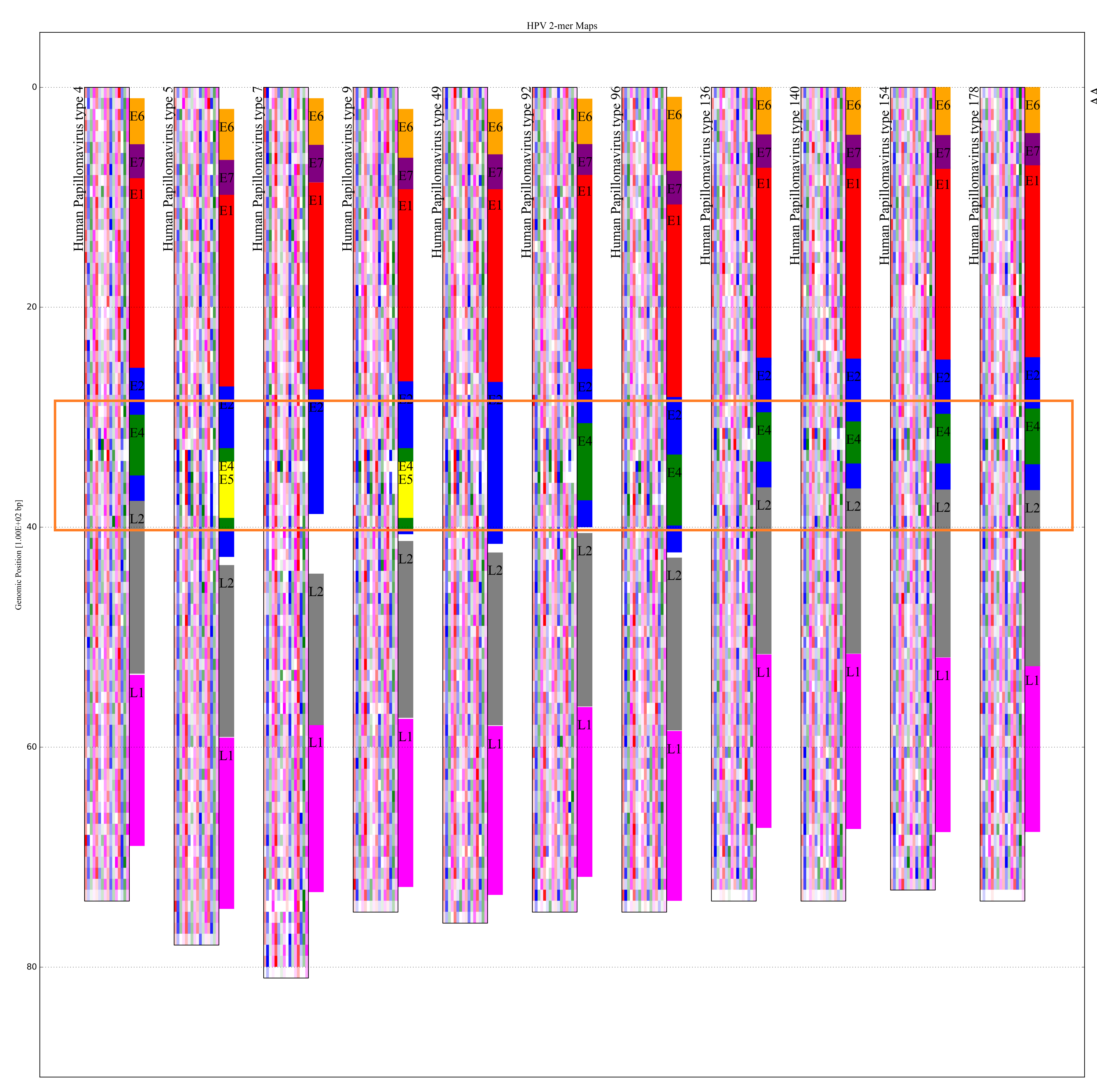

Supplement: Supplementary file 1 [file genes-08-00122-s001.zip › Fig. S1.png]

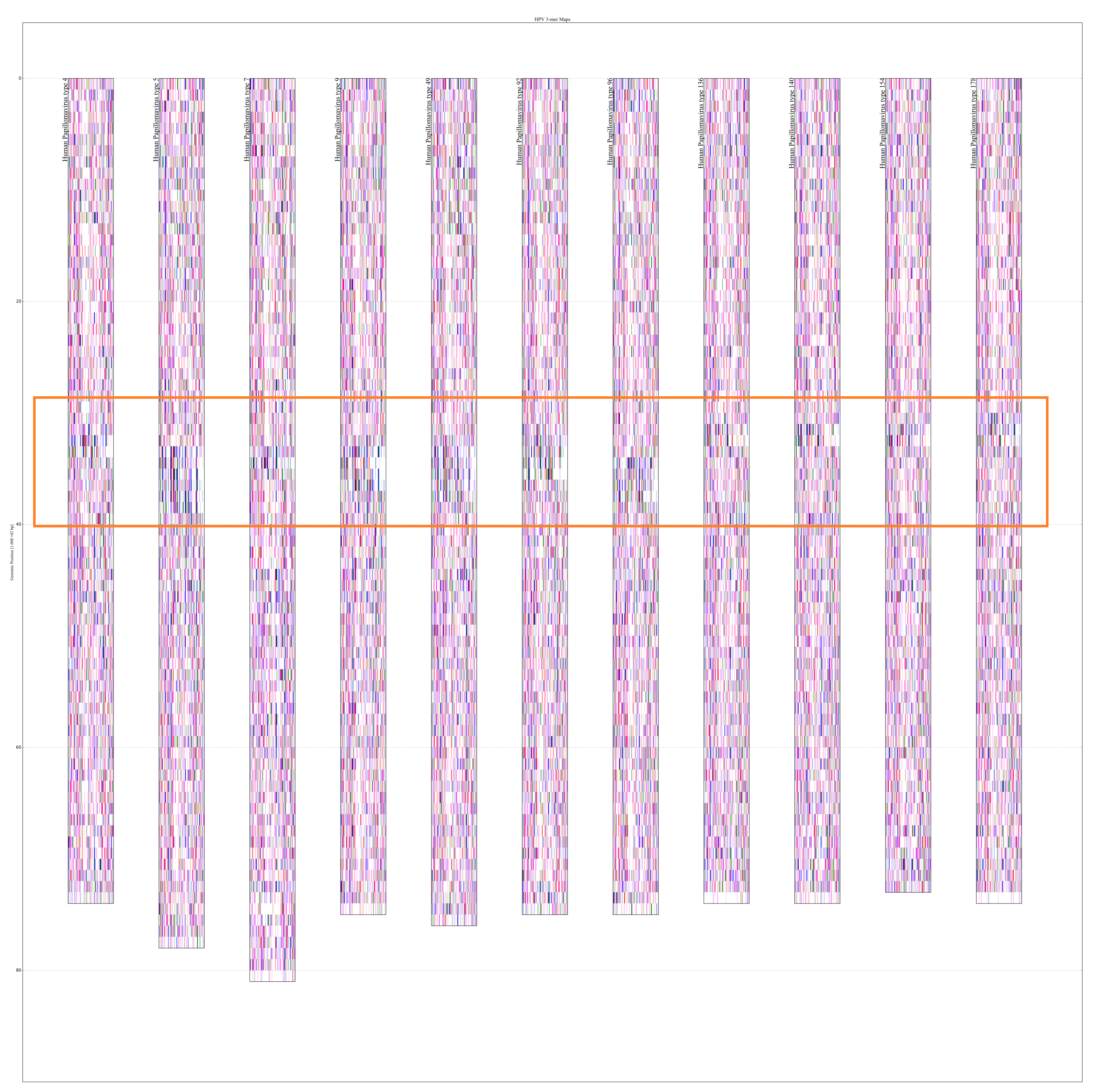

Supplement: Supplementary file 1 [file genes-08-00122-s001.zip › Fig. S2.png]

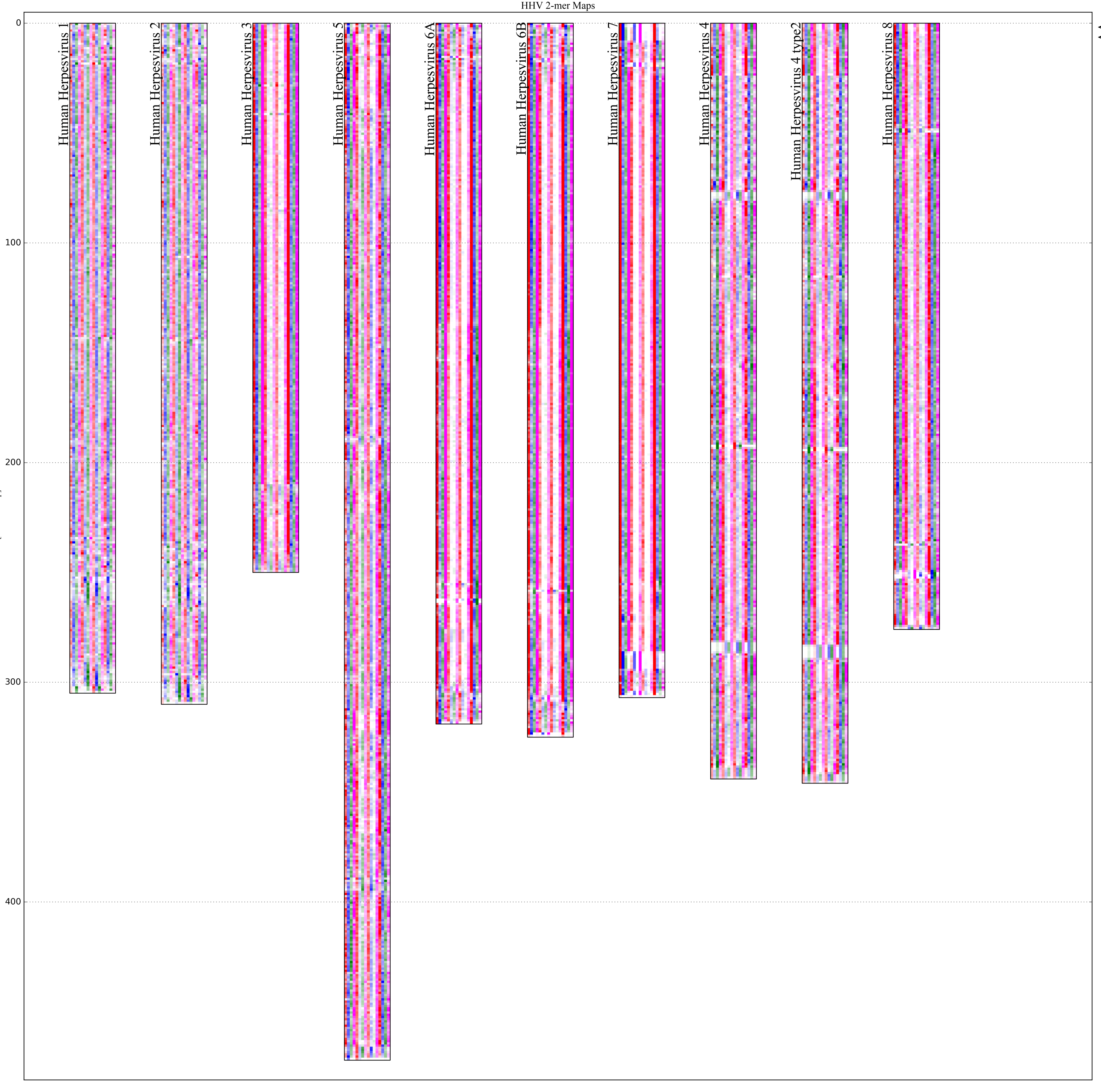

Supplement: Supplementary file 1 [file genes-08-00122-s001.zip › Fig. S3.png]

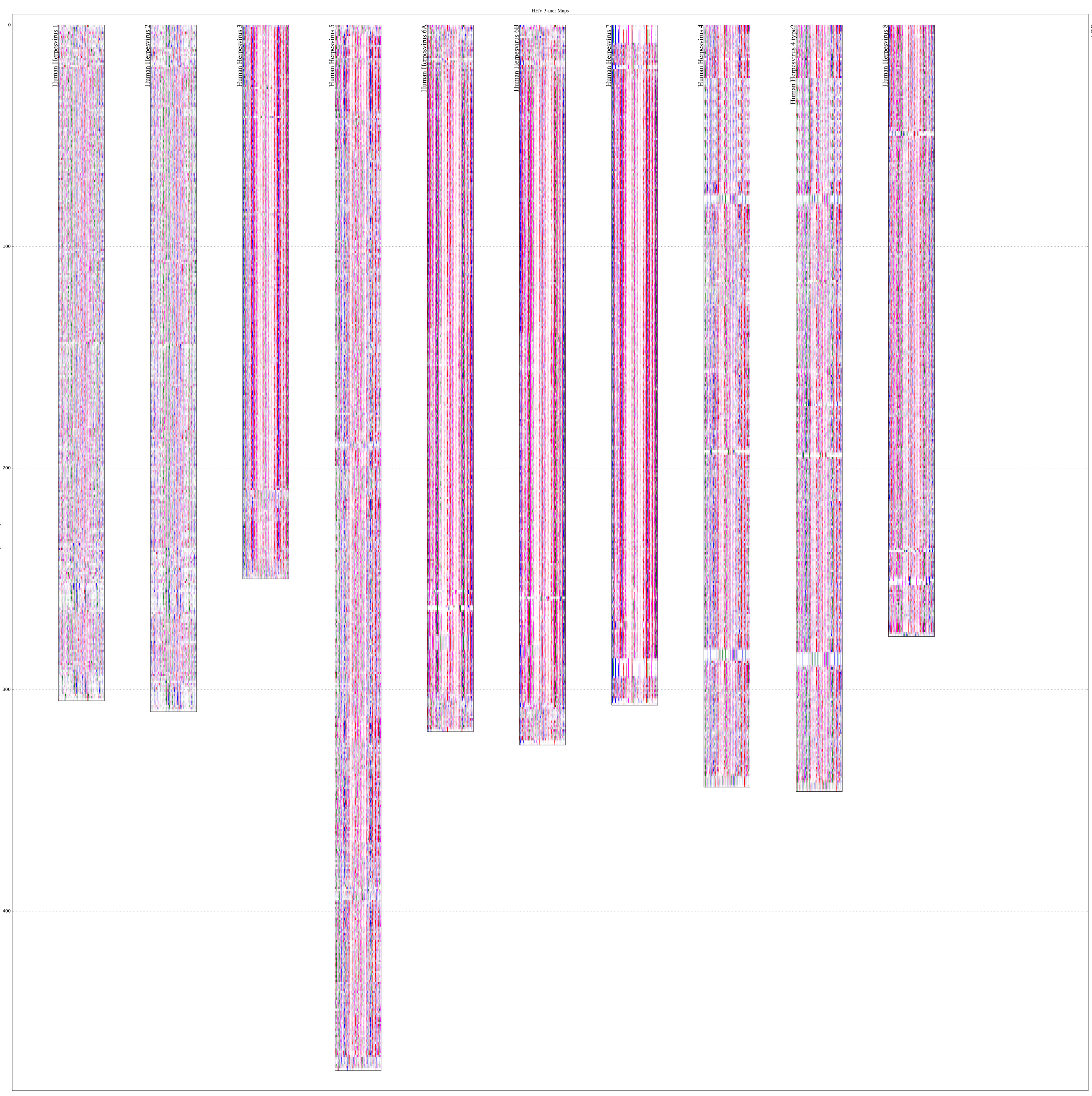

Supplement: Supplementary file 1 [file genes-08-00122-s001.zip › Fig. S4.png]
